# Supplementary material for: Deletion of wfs1 Impairs Oligodendrocyte Precursor Cells Dorsal Distribution and Myelination Through the wfs1-hmgcs1 Axis in Zebrafish
Source: Biology (Basel). 2026 Aug 16;15(16):1402. doi: 10.3390/biology15161402 (PMC13510151; doi:10.3390/biology15161402)
Supplement: Supplementary file 1 [file biology-15-01402-s001.zip › File S1-Transcriptome sequencing data and differentially expressed genes (DEGs) in wfs1-- zebrafish at 5 dpf/1.GO/wfs1KOvsWT/ALL/wfs1KOvsWT.all_GObar_bp.pdf]

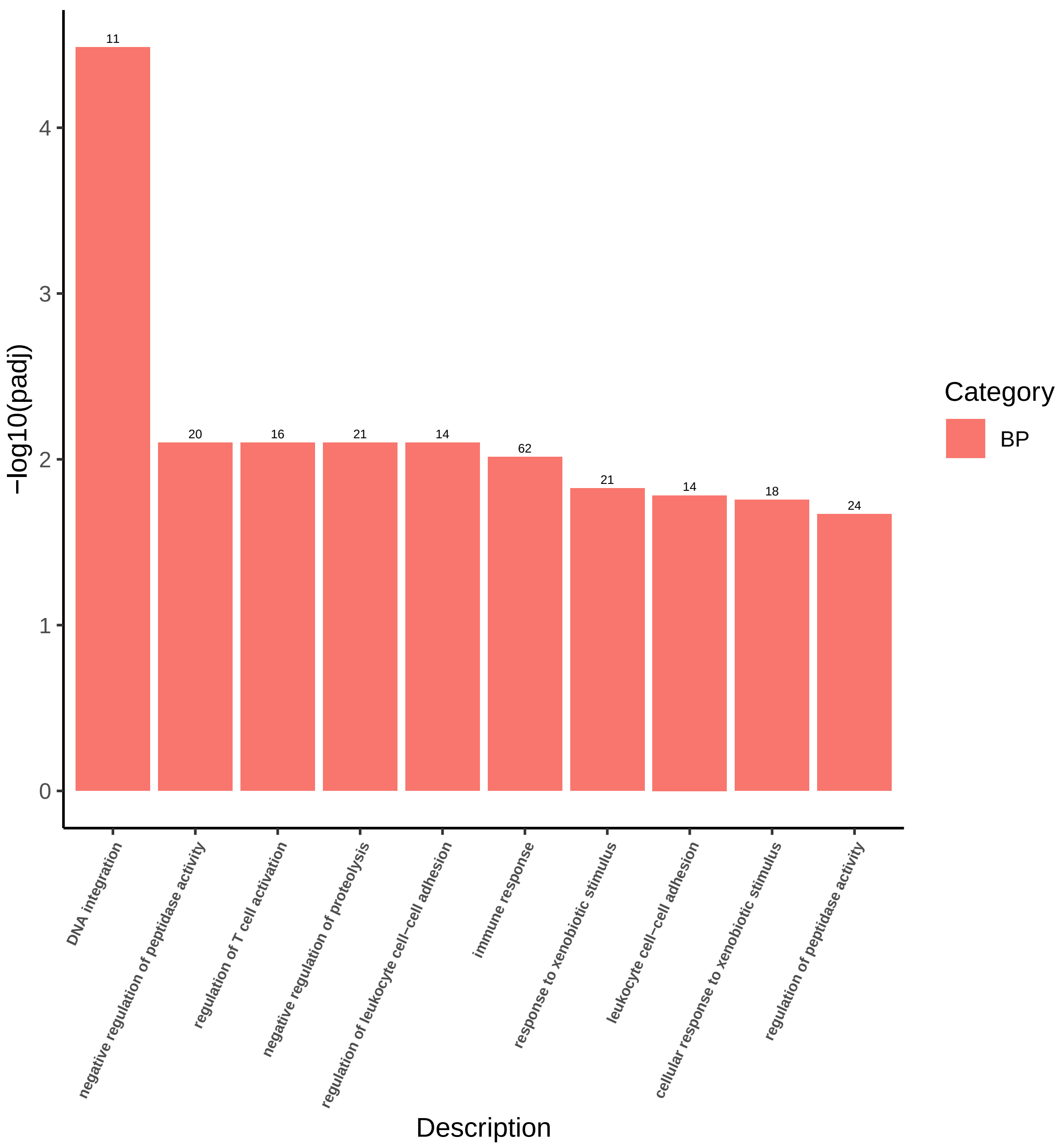

−log10(padj)
0
1
2
3
4
11
20
16
21
14
62
21
14
18
24
DNA integration
negative regulation of peptidase activity
regulation of T cell activation
negative regulation of proteolysis
regulation of leukocyte cell−cell adhesion
immune response
response to xenobiotic stimulus
leukocyte cell−cell adhesion
cellular response to xenobiotic stimulus
regulation of peptidase activity
Description
Category
BP
